# Molecular Research on Migraine: From Pathogenesis to Treatment

**DOI:** 10.3390/ijms24108681

**Published:** 2023-05-12

**Authors:** Antonino Tuttolomondo, Irene Simonetta

**Affiliations:** Internal Medicine and Stroke Care Ward, Policlinico University Hospital, proMISE Department, University of Palermo, 90127 Palermo, Italy; irene.simonetta@live.it

Migraine is a common, multifactorial, disabling, recurrent, hereditary, neurovascular headache disorder. Attacks often begin with warning signs (prodromes) and aura (transient focal neurological symptoms), of which the origin is thought to involve the hypothalamus, brainstem, and cortex. As a consequence of the disease itself or its genetic underpinnings, the migraine brain is altered structurally and functionally. These molecular, anatomical, and functional abnormalities provide a neuronal substrate for an extreme sensitivity to fluctuations in homeostasis, a decreased ability to adapt, and the recurrence of headache.

The diverse symptoms suggest that migraine is more than a headache. It is now viewed as a complex neurological disorder that affects multiple cortical, subcortical, and brainstem areas that regulate autonomic, affective, cognitive, and sensory functions. A major insufficiently understood issue in the neurobiology of migraine concerns the molecular and cellular mechanisms that underlie the primary brain dysfunction and lead to the activation and sensitization of the trigeminovascular system, thus generating and maintaining migraine pain.

This Special Issue aims to highlight some recent development in our molecular understanding of migraine and discusses novel opportunities for mechanistic studies. The invited authors will review recent discoveries that have advanced our understanding of these mechanisms toward a unifying pathophysiological hypothesis, in which cortical spreading depression (CSD), the phenomenon underlying the migraine aura, assumes a key role. In particular, the author discuss the significant contemporary findings in the genetics and neurobiology of some clinical subtypes of migraines, and the insights they provide into the molecular and cellular mechanisms that may lead to the increased susceptibility of CSD in migraineurs.

In this Special Issue of the *International Journal of Molecular Sciences*, authors have contributed with original studies or review manuscript to develop a more complete burden of knowledge about the molecular findings relating migraine, both by the means of pathogenetic and therapeutic tools.

Rubino et al. [1] analyzed the possible modulatory role of polymorphisms of the proinflammatory cytokine genes in the response to NSAIDs, but not to triptans in migraine attacks.

Considering that a significant percentage of patients does not obtain a satisfactory response to acute pain-relieving therapies, including NSAIDs and triptans, the authors analyzed the possible role of genetic influences on the therapeutic response to NSAIDs in migraine patients. Authors have reported that pharmacogenetics plays a key role in the understanding of such a diverse response. In order to investigate whether functional polymorphisms in proinflammatory cytokine genes (*IL-1α*, *IL-1β*, *IL-1RN*; *IL-6*, and *TNF-α*) may influence the response to acute treatment, authors studied 313 consecutive patients with episodic migraine without aura. Pain relief through the administration of NSAIDs or triptans for three consecutive migraine attacks was evaluated. Authors reported a significant association between the A allele of the *TNF-α* promoter (−308 A/G) and a lack of efficacy after NSAID administration compared with the G allele. The other analyzed polymorphisms demonstrated no significant effect on pain relief. This study showed that a functional polymorphism in the *TNF-α* gene can significantly influence the clinical response to NSAID administration in acute attacks. Patients with a higher production of the active cytokine during stress showed a significantly lower anti-migraine effect. These findings require further studies providing better knowledge of the a role of TNF-α in the pathophysiological mechanisms of migraine attack.

Nevertheless, the inflammatory pathogenesis of migraine attacks is worthy of further analysis, in order to identify the possible new inflammatory targets of anti-migraine attack treatments. 

Many researchers hypothesized that migraine could be a neurological disease in which the central nervous system (CNS) dysfunction plays a pivotal role, with the subsequent involvement of the trigeminovascular system, resulting in the expression of peripheral symptoms and aspects of pain [2].

The application of inflammatory substances on the dura mater, in addition to chemical stimulation of the dural receptive fields, causes hypersensitivity to mechanical and thermal stimulation, as well as activation in the trigemical ganglion (TG) and the brain stem trigeminal neurons, similarly to what could be expected in migraine. Thus, the modulation of inflammatory triggers involved in migraine pain attacks may also depend on the TNF-α cascade activation. 

In a further article, Porcaro et al. [3] analyzed the role of the hypothalamic mechanism regulating the duration of a migraine attack, with insights into the microstructural and temporal complexity of cortical functional network analysis. Since the reported involvement of the hypothalamus and the limbic system in the pathogenesis of the beginning of a migraine, the authors analyzed diffusion tensor imaging (DTI) parameters of the entire hypothalamus and its subregions in 15 patients during a spontaneous migraine attack and in 20 control subjects. They analyzed the non-linear measure resting-state functional MRI BOLD signal complexity using the Higuchi fractal dimension (FD) and correlated DTI/fMRI findings with patients’ clinical characteristics. Authors reported that compared to controls, the migraine patients showed significantly altered diffusivity metrics within the hypothalamus, mainly in posterior ROIs, and higher FD values in the salience network (SN). Authors also demonstrated a positive correlation of the hypothalamic axial diffusivity with migraine severity and FD of the SN. The DTI metrics of the bilateral anterior hypothalamus positively correlated with the mean attack duration. Migraine-related alterations in the sympathetic/parasympathetic function have a complex pattern; however, in general, an imbalance occurs between the sympathetic and parasympathetic tone. Through an improved understanding of the role of autonomic changes in the pathogenesis of migraine, it may be possible to develop more effective treatments for migraine sufferers. The findings of the study by Porcaro et al. further add to the information relating the autonomic nervous system (ANS) dysfunction in migraine headache sufferers.

In another review manuscript, Guerrero-Toro et al. [4] tested the role of glutamate NMDA receptors in peripheral trigeminal nociception implicated in migraine.

The pro-nociceptive role of glutamate in the CNS in migraine pathophysiology is well established. Glutamate, released from trigeminal afferents, provokes an activation of second-order nociceptive neurons in the brainstem. However, the function of peripheral glutamate receptors in the trigeminovascular system, suggested as the origin site for migraine pain, is less known. Authors evaluated calcium imaging and patch clamp recordings from trigeminal ganglion (TG) neurons, immunolabelling, CGRP assay and direct electrophysiological recordings from rat meningeal afferents to investigate the role of glutamate in trigeminal nociception. Under free-magnesium conditions, glutamate, aspartate, and, to a lesser extent, NMDA evoked calcium transients in a fraction of isolated TG neurons, indicating the functional expression of NMDA receptors. The fraction of NMDA-sensitive neurons was increased by the migraine mediator, CGRP. NMDA also activated slowly desensitizing currents in 37% of the TG neurons. However, neither glutamate nor NMDA changed the level of extracellular CGRP. TG neurons expressed both GluN2A and GluN2B subunits of the NMDA receptors. In addition, after the removal of magnesium, NMDA activated persistent spiking activity in a fraction of trigeminal nerve fibers in meninges. Thus, was indicated to glutamate activate NMDA receptors in somas of TG neurons and their meningeal nerve terminals in a magnesium-dependent manner. These findings suggest that peripherally released glutamate can promote the excitation of meningeal afferents implicated in the generation of migraine pain in conditions of inherited or acquired reduced magnesium blockage of NMDA channels, and support the usage of magnesium supplements in migraine. 

A recent report [5] elucidated the role of glutamate in migraine. A higher degree of glutamate expression in the brain, and possibly in the peripheral circulation, has been reported [6] in migraine patients, particularly during attacks. Altered blood levels of kynurenines, endogenous modulators of glutamate receptors, have been reported in migraine patients. Furthermore, genetics studies [7] implicate genes that are involved in glutamate signaling during a migraine, and gene mutations responsible for familial hemiplegic migraine and other familial migraine syndromes may influence glutamate signaling. Animal studies [8] indicate that glutamate plays a key role in pain transmission, central sensitization, and cortical spreading depression. This review by Guerrero-Toro et al. may furtherly underlines multiple therapies that target glutamate receptors, including magnesium, topiramate, memantine, and ketamine, that have been reported to have efficacy in the treatment of migraine; although, with the exception of topiramate, the evidence for the efficacy of these therapies is not strong. Moreover, because all of these therapies have alternative mechanisms of action, it is not possible to conclude that the efficacy of these drugs is entirely due to their effects on glutamate receptors. Further studies are needed to clearly delineate the possible roles of glutamate and its specific receptor subtypes in migraine, and identify new ways of targeting glutamate for migraine therapy.

In a study by Sharav et al. [9], the authors examined the possible differences between orofacial migraine (OFM) and neurovascular orofacial pain (NVOP). The facial presentations of primary headache are comparable to primary headache disorders, but occurring in the V2 or V3 dermatomes of the trigeminal nerve. These were classified and recently published in the *International Classification of Orofacial Pain, 1st edition* (ICOP). A category in this classification includes “orofacial pains resembling presentations of primary headaches,” which encompasses the OFM and NVOP. The differences between the NVOP and OFM are subtle, and their response to therapy may be similar. While classified under two separate entities, they contain many features in common, suggesting a possible overlap between the two. Consequently, their separation into two entities warrants further investigations. In this Editorial, we describe the OFM and NVOP, and their pathophysiology is discussed. The similarities and segregating clinical signs and symptoms are analyzed, and the possibility of unifying the two entities is debated.

Further, Simonetta et al. [10] examined the new insights into the metabolic and genetic pathogenesis of migraine, evaluating a possible therapeutic approach based on these mechanisms.

The authors underlined how according to electrophysiology and imaging studies, many brain areas are involved, such as the cerebral cortex, thalamus, hypothalamus, and brainstem. The activation of the trigeminovascular system has a key role in the headache phase. There also appears to be a genetic basis behind the development of migraine. Numerous alterations have been identified, and in addition to the genetic cause, there is also a close association with the surrounding environment, as though the genetic alterations may be responsible for the onset of migraine on the one hand, and the environmental factors seems to be more strongly associated with exacerbations on the other. This review is an analysis of the neurophysiological mechanisms, neuropeptide activity, and genetic alterations that have a fundamental role in justifying the best therapeutic strategy. To date, the goal is to create a therapy that is as personalized as possible, and for this reason, progress has been made in the pharmacological field in order to identify new therapeutic strategies for both acute treatment and prophylaxis.

Neuroinflammation has been implicated in the pathogenesis of migraine. In patients with migraine, peripheral levels of pro-inflammatory cytokines, such as interleukin-1β (IL-1β) and tumor necrosis factor-α, are known to be increased. Additionally, animal models of headache have demonstrated that immunological responses associated with cytokines are involved in the pathogenesis of a migraine. Furthermore, these inflammatory mediators may alter the function of tight junctions in the brain vascular endothelial cells in animal models, but not in human patients. Based on clinical findings showing elevated IL-1β, and experimental findings involving IL-1β and both the peripheral trigeminal ganglion and central trigeminal vascular pathways, the regulation of the Il-1β/IL-1 receptor type 1 axis may lead to new treatments for migraine. However, the integrity of the blood-brain barrier is not expected to be affected during attacks in patients with migraine. Yamanaka et al. [11] reviewed the neuroinflammatory mechanisms in migraine pathogenesis relating this issue. 

Finally, in their original article, Volubeva et al. [12] reported a single episode of cortical spreading depolarization increasing the mRNA levels of proinflammatory cytokines, calcitonin gene-related peptide and pannexin-1 channels in the cerebral cortex. Cortical spreading depolarization (CSD) is the neuronal correlate of migraine aura and the reliable consequence of acute brain injury. The role of CSD in triggering headaches that follow migraine aura and brain injury remains unclear. We examined whether a single CSD occurring in awake animals modified the expression of proinflammatory cytokines (Il1b, TNF, and Il6) and endogenous mediators of nociception/neuroinflammation-pannexin 1 (Panx1) channel and calcitonin gene-related peptide (CGRP), transforming growth factor beta (TGFb) in the cortex. A unilateral microinjury of the somatosensory cortex triggering a single CSD was produced in awake Wistar rats. Three hours later, tissue samples from the lesioned cortex, intact ipsilesional cortex invaded by the CSD, and homologous areas of the contralateral sham-treated cortex were harvested and analyzed using qPCR. Three hours post-injury, intact CSD-exposed cortexes showed increased TNF, Il1b, Panx1, and CGRP mRNA levels. The strongest upregulation of proinflammatory cytokines was observed at the injury site, while CGRP and Panx1 were upregulated more strongly in the intact cortexes invaded by CSD. A single CSD is sufficient to produce low-grade parenchymal neuroinflammation, with simultaneous overexpression of Panx1 and CGRP. The CSD-induced molecular changes may contribute to the pathogenic mechanisms of migraine pain and post-injury headache.

Migraine is an episodic headache disorder affecting more than 10% of the general population. A major insufficiently understood issue in the neurobiology of migraine concerns the molecular and cellular mechanisms that underlie the primary brain dysfunction and lead to the activation and sensitization of the trigeminovascular system, thus generating and maintaining migraine pain. Here, the author reviews recent discoveries that have advanced our understanding of these mechanisms toward a unifying pathophysiological hypothesis, in which cortical spreading depression (CSD), the phenomenon underlying migraine aura, assumes a key role. In particular, the genetics and neurobiology of migraine and the insights they provide into the molecular and cellular mechanisms were addressed, that may lead to the increased susceptibility of potential useful drugs in migraineurs. Another possible issue worthy of future addressing is the possible similarities between inflammatory pathogenesis of migraine and immunoinflammatory pathogenetic background of acute ischemic cerebrovascular syndromes (AICS) [13,14,15,16,17,18,19,20,21,22].
